# Management of choking in Motor Neuron Disease: patient and public involvement in a co-produced mixed-methods study protocol

**DOI:** 10.1186/s40900-026-00974-6

**Published:** 2026-09-17

**Authors:** Dorinda Moffatt, Lisa Hinton, Lucinda Spurway, Angela Martin, Neil Drinkwater, Donna Noonan, Claire Rose, Sara Mazzucco

**Affiliations:** 1 Prospect Hospice, Moormead Road, Swindon, SN4 9BY UK; 2 Nuffield Department of Primary Care Health Sciences, University of Oxford, Radcliffe Primary Care Building, Radcliffe Observatory Quarter, Woodstock Road, Oxford, OX2 6GG UK; 3 Swindon Community Speech and Language Therapy Services, HCRG Care Services Ltd, Downs Way, Swindon, SN3 6BW UK; 4 Motor Neurone Disease Association, Francis Crick House, 6 Summerhouse Road, Moulton Park, Northampton, NN3 6BJ UK; 5 Great Western Hospitals NHS Foundation Trust, Swindon, SN3 6BB UK; 6 Nuffield Department of Clinical Neurosciences, Wolfson Centre for Prevention of Stroke and Dementia, John Radcliffe Hospital, University of Oxford, Oxford, OX3 9DU UK

**Keywords:** Motor Neuron Disease, Choking, Disability, Communication difficulty, Patient and public involvement, Co-design, Co-production, Theory of change

## Abstract

**Background:**

Up to 70% of people living with Motor Neuron Disease (plwMND) report distressing episodes of choking (a feeling of suffocation or airways blockage) due to involvement of bulbar muscles. There is an evidence gap on management of choking in plwMND, which is mirrored by the absence of guidance and training for healthcare professionals. Choking in plwMND is associated with other signs of bulbar involvement, such as communication difficulties due to dysarthria, which can be a barrier to effective participation in research. In this study protocol, we describe an innovative approach to meaningful involvement of plwMND, to co-produce a research project on management of choking tailored to their needs and aligning with their priorities.

**Methods:**

Phase 1 (Patient and public involvement co-design meeting): We first engaged in a meeting with plwMND, carers and healthcare professionals, to identify their priorities in relation to improving management of choking in MND. To ensure inclusive participation, we used the Theory of Change framework; people with communication difficulties were supported by a Speech and Language Therapist through their chosen augmentative and alternative communication aids. A key priority identified was the need to produce evidence-based guidance for management of choking tailored to the needs of plwMND. Phase 2 (Healthcare research study protocol on management of choking): based on the outcome of Phase 1, a multidisciplinary team (including individuals with lived experience) designed a mixed-methods, multi-centre research project based on four work packages (WPs). WP1: systematic review of the existing scientific literature on management of choking; WP2: interviews and focus groups with people with lived experience of choking in MND; WP3: co-design of a clinical algorithm detailing interventions to manage choking, based on WP1 and 2; WP4: algorithm development and dissemination with continuous involvement and input from people with lived experience through a Patient and Public Involvement co-applicant and patients’ representatives.

**Discussion:**

In plwMND and choking, barriers to inclusion in PPI and research range from reduced accessibility due to physical disabilities, to discriminatory sampling due to communication issues. Through inclusive engagement at the outset, plwMND highlighted a mismatch between their priorities (gaining knowledge about choking and feeling empowered to manage it at home) and available evidence (lack of guidance and training for healthcare professionals). This research project aligns with what matters to most affected patients, their carers and healthcare professionals, aiming to fill an evidence gap through new guidance to manage choking tailored to their needs.

**Supplementary Information:**

The online version contains supplementary material available at https://doi.org/10.1186/s40900-026-00974-6.

## Abstract (plain language summary)

Motor Neuron Disease (MND) is a life-limiting condition that damages nerve cells in the brain and spinal cord. It gets worse over time and cannot be cured. As the disease progresses, muscles become weaker, including those used for breathing, swallowing, coughing and speaking. People with MND often have trouble keeping their airways clear. Food, drinks or saliva can go down the wrong way and block the airway. This can cause choking. In people with MND, choking can happen more often, be more severe, and last longer than in other people. It can also happen outside of mealtimes. Choking is frightening for both patients and carers. It can lead to emergency calls, hospital visits and urgent medical treatment. Knowing what to do during a choking episode is important for patients, carers and healthcare professionals. However, there is currently no specific guidance or training designed for people with MND and those who care for them. Communication can also become difficult for people with MND, which can make it harder for them to take part in research and have their voice heard. In this project, people with lived experience of MND and episodes of choking have worked together to identify new research outcomes and set the path for next stages of research in this field. Together we will: (1) review and summarise the best available evidence on how to manage choking in MND, and identify gaps in knowledge; (2) talk to people with MND, their carers and healthcare professionals, to understand their experiences and needs; (3) use this information to develop practical guidance on how to manage choking; (4) share this guidance with the MND community and healthcare professionals.

**Supplementary Information:**

The online version contains supplementary material available at https://doi.org/10.1186/s40900-026-00974-6.

## Background

Motor Neuron Disease (MND) is a life-limiting condition with no cure, where symptom management has a central role to optimise quality of life. It causes severe physical, cognitive and psychological disability with significant emotional burden on people living with MND (plwMND) and their carers [1, 2].

MND causes clinically significant bulbar involvement in at least 80% of patients [2]. Degeneration of pathways innervating pharyngeal, laryngeal and lingual muscles results in dysarthria, dysphonia and dysphagia; involvement of respiratory muscles causes weak cough and reduced airway clearing, making plwMND prone to episodes of prolonged coughing and choking (a feeling of suffocation, breathing impediment or airways blockage) [3, 4]. Choking is a common complaint in the clinical environment and is reported by up to 70% of plwMND [3]. While in the general population coughing and choking are distinct phenomena, they are difficult to differentiate in plwMND, where they are more frequent, prolonged and severe [3]. Choking in plwMND is also caused by ‘laryngospasm’ due to vocal folds dysfunction. Laryngospasm in MND has been reported in the neurological and otolaryngology literature with a frequency varying between 2% and 30% [4, 5]. Although laryngospasm is associated with acute dyspnoea and a feeling of choking, it seems to be independent of respiratory function and has been described in other neurological conditions such as Multiple System Atrophy, Parkinson’s Disease and stroke [4, 5].

Secretions management is also challenging in plwMND, intended both as excessive saliva and drooling, and as thick mucus accumulation, which are associated with fear of choking [6, 7].

Choking is intensely distressing for both plwMND and their carers, not least due to a perceived incumbent risk of aspiration and death [3, 8]. Although anxiety can worsen these episodes, there is no evidence that a history of anxiety increases number, severity, or duration of coughing and choking episodes [3]. However choking is anxiety-generating, adding further to the psychological burden of plwMND and their carers [3, 8]. This is particularly important as psychological distress is associated with shorter survival time and increased risk of suicide in plwMND [9].

Despite being highly distressing, choking is a neglected symptom in MND literature [3–5]. Evidence on best treatment for choking is lacking, and guidance for management of choking specifically developed around the needs of plwMND is missing. The National Institute for Health and Care Excellence (NICE) guidelines on MND mention ‘breathlessness’ rather than ‘choking’ and fail to give clear indication about treatment [10]. They only suggest benzodiazepines and opioids to manage breathlessness exacerbated by anxiety, and do not provide details about which specific drugs should be used, their dose, formulation, route and timing of administration [10]. These are all critical issues in people with bulbar symptoms causing swallowing impairment or having a feeding tube in place. Another relevant guidance recommends plwMND experiencing ‘any problems with swallowing, coughing or choking, (to) see a speech and language therapist and a dietitian for advice’ [11]; however, there is no specific training available for healthcare professionals, tailored to choking in MND; guidelines are based on the general population who can cough effectively or stand up to receive back blows [11, 12].

Risk of choking in plwMND goes together with communication difficulties, as patients progressively develop bulbar symptoms and dysarthria, up to complete anarthria - which leads them to ‘lose their voice’, both physically and metaphorically. Neurological disabilities are among the most frequent barriers to research participation [7, 13, 14]. Due to how disability is defined and quantified, communication disability is often under-measured and under-represented, both in healthcare services design and in research [15, 16]. People with a high burden of disability both on a physical and communication level, such as plwMND, may represent a group at particularly high risk of being ‘unheard’ and not appropriately involved in designing healthcare services and research, where they are the main stakeholders [17]. The importance of involvement of people with communication disabilities in both is now not only recognised, but set as a recommendation by the World Report on Disability, last issued in 2011 [18].

A way to facilitate meaningful patient and public involvement (PPI) is through the Theory of Change; this is an approach to planning change for a group or community, detailing how to achieve specific long-term outcomes through a sequence of shorter-term outcomes, which are identified through a backward-mapping approach [19]. While it is used in many fields to support strategic planning, monitoring and outcomes evaluation, its use in research is still limited [20, 21]. Its democratic approach lends itself to shape meaningful involvement of stakeholders in research, promoting outcomes relevance, ownership and reducing power imbalance [22].

This paper reports on two aspects of our work: the PPI process (Phase 1) that led to the co-design of the study protocol ‘Management of choking in people living with Motor Neuron Disease: a co-designed, evidence-based clinical algorithm’, (Phase 2), which describes an innovative approach to meaningful involvement in healthcare research of people with lived experience of MND, choking and communication disability.

## Methods

**Fig. 1:**
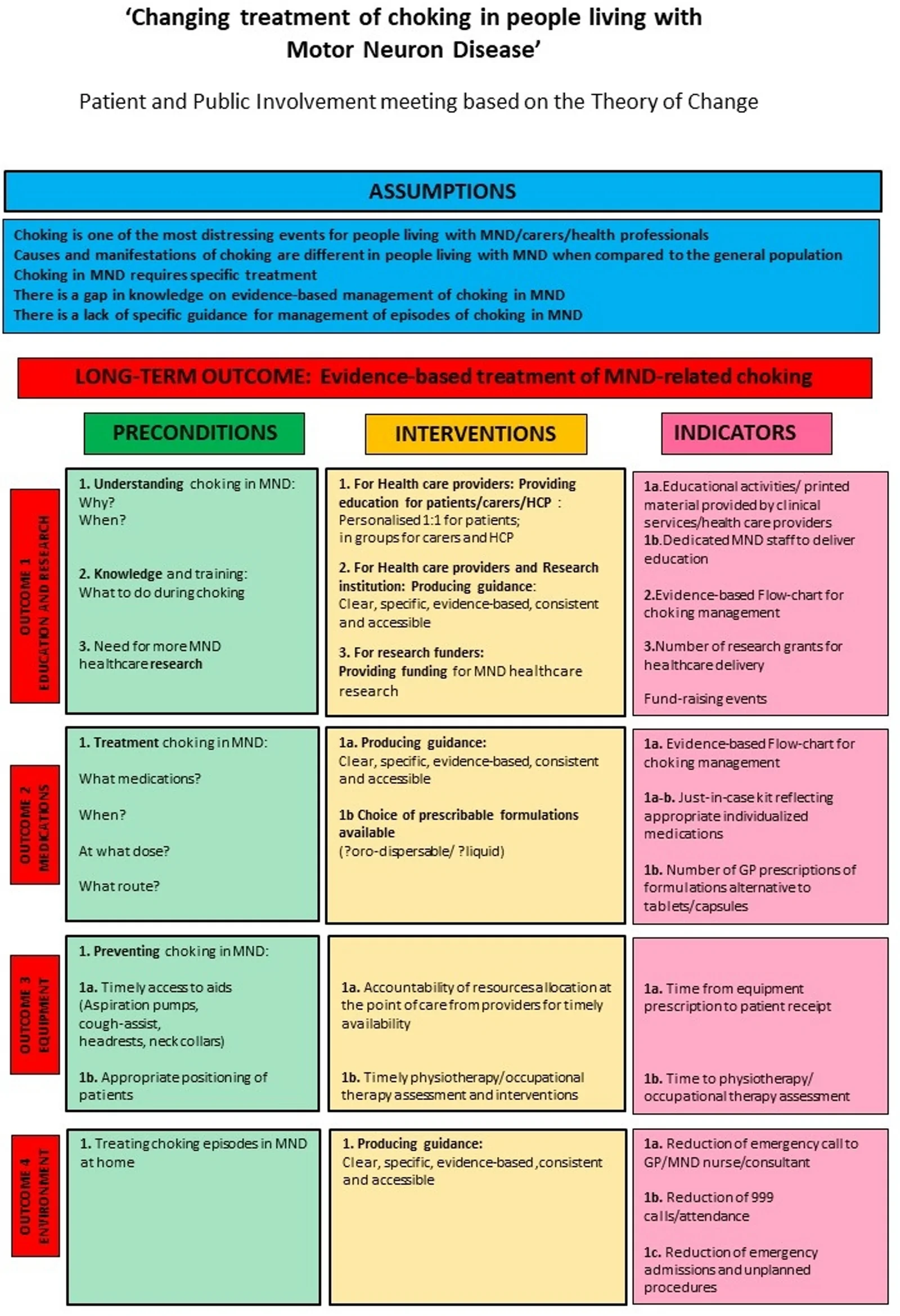
Outcome of the patient and public involvement meeting using the theory of change framework

At the initiation of the project (Phase 1), we engaged in a PPI meeting with plwMND, carers and healthcare professionals, to co-design the ‘pathway of change’ to improving management of choking in MND (‘co-design meeting’) (see below and Fig. 1). Based on the outcome of the co-design meeting, we then actively engaged with them as co-researchers in a research project, the rationale and protocol of which is detailed in the ‘Healthcare research study on management of choking’ section (Phase 2).

### Phase 1 - Patient and public involvement co-design meeting

PPI input was designed according to the GRIPP2-SF guidance [23].

### Aims

The aim of the PPI meeting was to co-design, with representatives of all the relevant stakeholders, the ‘pathway of change’ to improve management of MND-related choking.

### Methods

#### Patients and setting

For the PPI meeting we invited 18 people with lived experience of MND and choking, purposively sampling plwMND, carers, healthcare professionals and research funders [24]. The invitation letter, the meeting schedule, and details of the meeting can be found in the Supplementary Material.

The meeting was held on February 10th 2024 at Prospect Hospice in Swindon (UK) and was led by a consultant neurologist with a special interest in MND (SM).

Fifteen people overall took part (Supplementary Material page 8). Three healthcare professionals attended; one MND and Neurorespiratory Specialist Practitioner, DM, one healthcare assistant with an interest in MND, and one Specialist Speech and Language Therapist, LS, who supported attending plwMND with communication difficulties through their chosen augmentative and alternative communication aids. Two plwMND attended; one was a 47-year-old man with advanced MND (Participant A), quadriplegia and anarthria, who came on his electric wheelchair with a headrest, accompanied by his wife. He used his own head-mouse operated speech generating device during the meeting; LS helped with the set-up of his communication aid and supported him throughout the meeting to allow him full participation. The second patient (Participant B) was an 80-year-old man with a bulbar form of MND and moderate dysarthria; he came wearing his head-up collar accompanied by his wife, two daughters and one son in law. The remaining participants were three family carers of two deceased patients who had experienced episodes of choking, and a representative of the MND Association.

#### Theory of change framework

We used the Theory of Change as an inclusive and democratic framework to engage all stakeholders in the co-design meeting [22]. The Theory of Change uses a mapping process which starts by identifying the desired long-term outcome and then maps backward the required ‘pathway of change’. This breaks down the long-term outcome into sets of more specific outcomes. For each, a number of ‘preconditions’ is defined, which are intended as the ‘ingredients’ that the group thinks would be necessary to achieve the long-term outcome. For each outcome, several ‘Interventions’ are identified, each in turn with appropriate ‘Indicators’ as measures of the achieved change [19, 20].

Participants A and B indicated that they preferred for the discussion to pause and allow them time to formulate their contribution.

#### Outcome of the meeting

The outcomes of the meeting are summarised in Fig. 1 and detailed in the Supplementary Material. In summary, the ultimate long-term outcome set by stakeholders was for plwMND, carers and healthcare professionals ‘to be able to manage choking episodes in an evidence-based way’. According to the Theory of Change, this broad outcome was broken into overarching sets of more specific outcomes (Fig. 1, Outcomes 1,2,3,4); for each, a few ‘Preconditions’ were defined. Several ‘Interventions’ were identified for each outcome, with appropriate ‘Indicators’ as a measure of the achieved change. As part of outcome 1 (Education and research), MND healthcare research aiming to produce ‘Clear, specific, evidence-based, consistent and accessible guidance’ for management of choking episodes was identified as an intervention to be actioned by healthcare providers, funders and research institutions. The related indicator was ‘production of an evidence-based flow-chart for management of choking in MND’. The need for such guidance emerged also as part of Outcome 2 (Medications) and 4 (Treating choking episodes in MND at home).

Participant B was particularly vocal on the need for research in the field of MND-related choking; one of his family members stated that, ideally, guidance for managing of choking episodes *‘should be laminated and hung on the fridge.’*

There was agreement that the best format for such guidance would be a flow-chart. An ‘Evidence-based flow-chart for choking management’ was therefore chosen as indicator for both Outcome 1, 2 and 4. In outcome 4, there was unanimous agreement that plwMND and their carers should be able to treat choking at home, without anxiety-generating emergency calls. Participants A and B agreed that their wish was to be kept out of hospital and to be empowered to treat choking episodes at home. This was also felt would be more sustainable and benefit the NHS, sparing emergency consultations, ambulances attendance, and hospital admissions.

Feedback was collected on how patients and carers would prefer to be involved in further research on management of choking. Patients indicated a preference for individual interviews in a flexible way (remotely or face-to-face) while carers and healthcare professionals agreed on focus groups.

## Phase 2 - Healthcare research study protocol on management of choking

### Study design and methodology

**Fig. 2:**
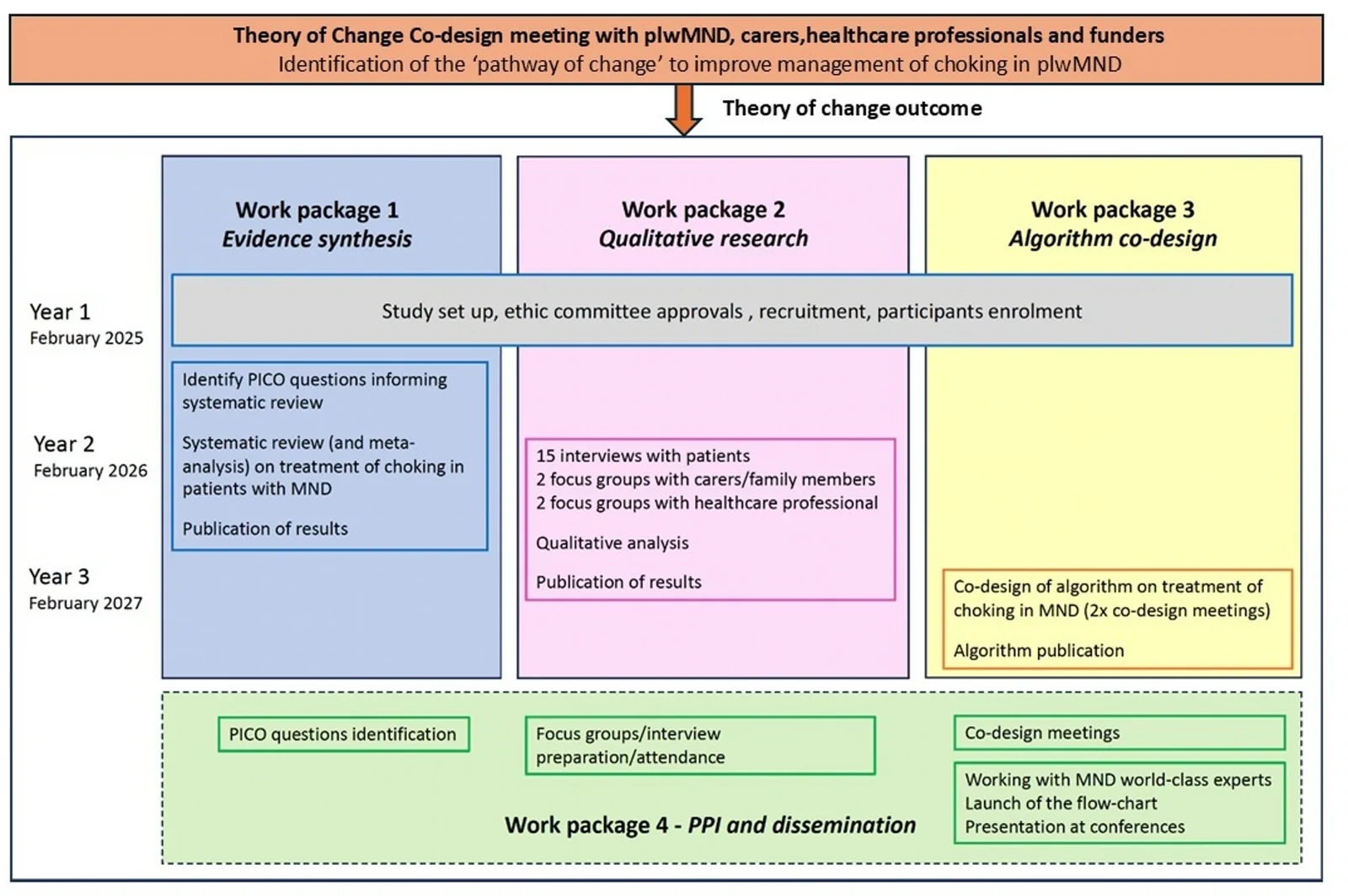
Outline of the ‘pathway of change’ leading to the research project: “Management of choking in people living with Motor Neurone Disease: a co-designed, evidence-based clinical algorithm”

Based on the outcome of the PPI meeting and given the upcoming opportunity to apply for funding within the ‘Healthcare’ research stream of the MND Association, we sought to co-produce, with people with lived experience as co-researchers, a research project entitled ‘Management of choking in people living with Motor Neurone Disease: a co-designed, evidence-based clinical algorithm’. This is a mixed-methods study integrating an evidence synthesis component and a qualitative one, overall articulated in four work packages (WPs, Fig. 2):

WP1: review and synthesise the existing evidence on management of choking through a systematic review of available interventions.

WP2: interviews with plwMND and focus groups with their carers and healthcare professionals, to gather their experiences and views on how choking is managed in MND and what support they need.

WP3: involvement of plwMND, carers and healthcare professionals in the co-design and development of a clinical flow-chart (algorithm), based on the findings of WP1 and 2, detailing interventions for management of choking.

WP4: continuous involvement and input from people with lived experience of MND through a PPI co-applicant and a patients’ representative in the research team; dissemination of the algorithm to plwMND, their carers, healthcare professionals and the wider scientific community.

For the purpose of this study, we defined as ‘co-design’ the collaborative, creative approach incorporating experiences, values and priorities of stakeholders (human-centred) in the development of the research project and of the algorithm to manage choking in MND. For example, informing participants’ recruitment and engagement, or the operational definition of ‘choking’. ‘Co-production’, on the other hand, referred to the nature of the contribution of lived experience co-researchers, with shared decision-making and active participation throughout the research, including data collection, interpretation and dissemination [25].

The project was awarded a grant from the Motor Neurone Disease Association in December 2024 (Mazzucco/Oct24/2410 − 794).

#### Definition of ‘choking’

The input of experts by experience during the PPI meeting (Phase 1) and of those involved as co-researchers in the project (Phase 2), shaped a more inclusive definition of ‘choking’, beyond its common meaning of ‘airway obstruction’. It included a spectrum of symptoms describing pharyngeal discomfort related to choking, laryngospasm and, more generally, laryngeal hypersensitivity, such as coughing, uncontrollable coughing, gagging, retching and ‘foreign body feeling’. This extended the scope of the search strategy for the systematic review and shaped the qualitative research, reflecting the wide range of experiences of plwMND and bulbar symptoms.

#### WP1: Evidence synthesis (Systematic review)

The protocol of the systematic review was written in collaboration with people with lived experience, carers and healthcare professionals looking after plwMND. Their input shaped the details of participants, interventions, comparators, and outcomes (PICO) and the search strategy [26, 27], and they have been involved in interpreting the review results. The protocol of the systematic review is registered on PROSPERO (CRD420251023239), the data collection has been completed and we will publish the results in a peer reviewed journal.

#### WP2: Qualitative research

The qualitative component of the study is in progress, and is intended as an iterative process. We will purposively sample plwMND, carers and healthcare professionals from across multiple collaborating centres so as to capture rich experiential data about choking in plwMND and its management. We will sample people with middle and late stage MND (from moderately disabled to severely disabled, King’s stage 2–4), and for diversity in terms of sex, ethnicity, socio-economics, geography, age, family context, length of time living with (or caring for someone with) MND and cognitive performance. Patients with mild cognitive impairment with capacity to consent will not be excluded.

For staff participants, a sampling framework will include age, sex, ethnicity, geography and length of time working as a professional to provide care for plwMND. Fifteen in-depth interviews with plwMND have been planned, with a data saturation approach. Two focus groups will be carried out with carers and two with healthcare professionals, each with six to eight participants. If we are unable to recruit sufficient numbers to focus groups due to participant availability, we will offer individual interviews. Patients and carers participants will be recruited through the NHS and participating hospices via MND coordinators/specialist nurses in MND services from Swindon, North Wiltshire, Gloucestershire, Bath/North-East Somerset, Devon and London. A topic guide for interviews and focus groups has been developed using evidence from WP1 and in discussion with the PPI co-applicant and patient representatives. Following informed consent, interviews and focus groups will be audio recorded for transcription to facilitate analysis. Data collection and analysis [28, 29] is being led by an experienced qualitative researcher from the Oxford University’s Nuffield Department of Primary Care Health Sciences (LH) with the contribution of a clinical member of the research team, to ensure clinical details are fully captured.

#### WP3: Intervention co-design

WP3 will focus on the co-design of an algorithm (clinical flow-chart) with specific and detailed guidance for the management of choking in plwMND [30]. Participants will be involved in two co-design workshops to iteratively develop the algorithm. Each workshop will include 4 plwMND and 4 carers, 2 MND healthcare professionals and 1 MND Association representative. Sampling will be as for WP2 but will exclude those who have participated in WP2. Results of the systematic review (WP1) and the qualitative research (WP2) will inform WP3. The outcomes and key themes/touchpoints to emerge from WP1 and WP2 will be presented during the first co-design meeting to prompt discussion on key elements for inclusion in the algorithm. Based on the feedback obtained, a prototype of the algorithm will be drafted, and this will then be presented at a second co-design meeting for further input and refinement. Additional changes will be agreed upon at the second meeting, before the algorithm is finalised.

To make this process accessible for plwMND and to ensure all perspectives are heard, we may need to adapt this sequence of events according to the capabilities of the patient participants, for example, through staged input with several smaller sessions. We will avoid hybrid meetings, and conduct each meeting either wholly remotely of face-to-face, depending on the characteristics of the group.

#### WP4: Public and patient involvement and dissemination

This project is co-produced with people with lived experience of MND, involving plwMND and carers. To promote PPI ownership and involvement, patients’ representatives are included in the research team, and one of the research co-applicants and WP4 lead is a PPI representative, spouse of a deceased MND patient (CR). They will be the guarantor of PPI input at each phase of the project (see Fig. 2). The WP4 team will also oversee the dissemination processes for the study.

### Qualitative analysis

Interviews and focus groups will be transcribed verbatim. Recordings, transcripts and fieldnotes will be de-identified and uploaded to a qualitative data analysis software programme (e.g. NVivo). Data will be analysed using a thematic approach to identify key themes and touchpoints for use in WP3 co-design. Analysis will be led by an experienced qualitative researcher from Oxford University’s Nuffield Department of Primary Care Health Sciences (LH). The co-design meetings will be audio-recorded for minute taking and reporting [29, 30]. The qualitative component of the project received ethical approval in December 2025 (IRAS 360194; REC 25/PR/1142).

## Discussion

We describe a novel approach to meaningful PPI in healthcare research aiming to improve management of choking in MND. There are many key innovative elements to this approach [31].

We included from the outset (Phase 1) people with severe physical and communication disability, who are at highest risk of being excluded from research [13, 15, 17]. Evidence shows that PPI in research is at best patchy and often tokenistic [32, 33]. For people with neurological disabilities, barriers to inclusion range from reduced accessibility due to physical disabilities, to discriminatory sampling due to communication issues. By affecting both mobility and communication, MND puts affected people at particularly high risk of exclusion from research [31, 34]. To ensure that plwMND and choking were not excluded due to concomitant communication difficulty, we supported participants with dysarthria/anarthria to use their chosen augmentative and alternative communication aids, through a Speech and Language Therapist, as recommended by the last World Report on Disability [18]. The voice of these most affected patients was included from the outset, during the PPI meeting, using the Theory of Change as a flexible and inclusive framework to draw the ‘pathway of change’ to improving management of choking in MND [19–21]. Themes emerging from a general discussion, paced around the needs of involved plwMND, highlighted a mismatch between patients’ priorities (gaining knowledge about choking and feeling empowered to manage it at home) and available evidence (lack of guidance and training for healthcare professionals). The Theory of Change framework brough this misalignment into focus and allowed the team to identify the production of new evidence on management of choking in MND as a priority, exemplifying how clinical research should be as inclusive as possible from the outset.

As a testimony of the positive impact of PPI in research co-design, the research project (Phase 2), stemming from the initial PPI meeting, secured a three-year funding award from the MND Association through a competitive, peer reviewed process, and is currently ongoing. This is an example of co-design and co-production, ensured by involvement of a PPI co-applicant with lived experience of MND and choking, and by inclusion of patients’ representatives in the research group. The co-researchers have contributed to every step of the project, from the choice of the project title, to the study design and grant application; they have been involved in the development of the evidence synthesis by identifying the PICO questions of the systematic review, the search strategy and interpreting the results. They have contributed to developing the documentation for ethical approval, to engage participant identifying centres, collect data and interpret them. They are contributing to the dissemination of the project at each stage, which guarantees ongoing feedback from the relevant stakeholders.

The outcome of the project, a clinical algorithm to manage choking, will be a material example of intervention co-design in palliative care research, that can be applied to other clinical areas and chronic conditions. The algorithm is expected to improve delivery of care to plwMND by alleviating anxiety around choking, increasing quality and consistency of advice from healthcare professionals, optimising ad-hoc medications and reducing the need for emergency calls and unplanned hospital admissions in cases of choking – with gains for plwMND, carers and healthcare providers.

There are some potential limitations to our approach. First, we have conducted only one PPI co-design meeting; all participants were of Caucasian ethnicity and attending plwMND were both male; iteration could have brought more breadth and diversity to the initial phase of the research. However, the meeting was attended by people with a wide range of lived experience and was shaped by the needs of the most vulnerable plwMND, who were able to fully express their views. Secondly, despite emerging evidence on potential applications of the Theory of Change for community-led approach to healthcare services development and improvement [35], there is not much published evidence on its use to inform design of healthcare research [20–22]. While this contributes to the novelty of this project, there is no set way to assess its outcome and the quality of the process. Thirdly, the ultimate output of the project, the clinical algorithm to guide management of choking in MND, might come with challenges and barriers that are currently difficult to predict. Although it aims to fill the gap in current clinical guidelines, where we have identified a misalignment between what matters to patients and what evidence is available in the scientific literature, the algorithm will need to be validated and regularly updated in light of new evidence becoming available.

In conclusion, in this paper we describe an innovative approach to meaningful PPI in healthcare research, from study co-design to intervention co-production, to address an evidence gap and unmet need, and improve care for some of the most vulnerable plwMND, who are at risk of not having their voice heard.

## Supplementary Information

Below is the link to the electronic supplementary material.

Supplementary Material 1

## Data Availability

No datasets were generated or analysed during the current study.

## Abbreviations

MND: Motor Neuron Disease
plwMND: People living with MND
WP: Work Package
NICE: National Institute for Health and Care Excellence
PPI: Patient and public involvement
PICO: Population, interventions, comparators, and outcomes
NHS: National Health System
