# Supplementary material for: Management of choking in Motor Neuron Disease: patient and public involvement in a co-produced mixed-methods study protocol
Source: Res Involv Engagem. 2026 Sep 17;12:147. doi: 10.1186/s40900-026-00974-6 (PMC13584399; doi:10.1186/s40900-026-00974-6)
Supplement: Supplementary file 1 [file 40900_2026_974_MOESM1_ESM.docx]

**Management of choking in Motor Neuron Disease: patient and public involvement in a co-produced mixed-methods study protocol.**

Moffatt D,^1^ Hinton L,^2^ Spurway L,^3^ Martin A,^2^ Drinkwater N,^4^ Noonan D,^5^ Rose C, Mazzucco S^5,6^

^1^ Prospect Hospice, Moormead Road, Swindon, SN4 9BY

^2^ Nuffield Department of Primary Care Health Sciences, University of Oxford

^3^ Swindon Community Speech and Language Therapy Services, HCRG Care Services Ltd

^4^ Motor Neurone Disease Association

^5^ Great Western Hospitals NHS Foundation Trust

^6^ Wolfson Centre for Prevention of Stroke and Dementia, Nuffield Department of Clinical Neurosciences, University of Oxford

**Appendix:**

**Patient and Public Co-design Meeting**

Letter of invitation………………………………………………………………………… Page 3

Meeting agenda……………………………………………………………………………. Page 5

Consent to audio recording……………………………………………………………. Page 6

Feedback Form…………………………………………………………………………….. Page 7

Participants………………………………………………………………………………….. Page 8

Meeting report…………………………………………………………………………….... Page 9

MND association

PROSPECT Hospice

The Great Western Hospital NHS Foundation Trust

**Patient and Public Involvement meeting on:**

**‘Treatment of choking in people living with Motor Neurone Disease:**

**what is missing?’**

***February 10^th^ 2024 10.30 am to 12.30 pm***

***Prospect Hospice, Moormead Road, Wroughton, Swindon SN4 9BY***

You are receiving this invitation because you have been identified by our team as someone living with Motor Neurone Disease (MND), or you are caring, or have cared, for someone with MND.

Our team is planning a **research project** **on the topic of choking in MND**. Coughing and choking episodes are more frequent, severe, and prolonged in people living with MND than in the general population and can happen outside of meals. They are **distressing for patients and carers**, and can lead to emergency calls, hospital admissions and unplanned medical procedures.

Current protocols for treatment of choking are based on data from the general population, and there are no guidelines specific to people living with MND. **We want to fill this gap**. In order to do this, we need to understand what is most important to patients and carers when it comes to the treatment of choking. By working with you, our aim is to develop evidence-based guidance for the treatment of choking in people living with MND.

We would be very pleased if you could join our Patient and Public Involvement meeting for people living in the Swindon area on the subject of ‘*Treatment of choking in people living with Motor Neurone Disease: what is missing?*’ In this meeting, we hope **local people with lived experience of MND**, their **carers** and **loved ones**, **will tell us** **what, in their view, is missing in the way choking is managed.** Based on your ideas and experience, we will work out what matters the most about treating choking, what is missing in the current way choking is treated, and what we need change. **Your experience, views, values and priorities will inform our research project**, helping us to understand how management of choking can be improved.

*If you are experiencing changes in your speech because of the disease, it is even more important that you attend*; your point of view really matters to us and can be shared in whichever way you prefer, so if you have one, please bring your usual communication aid. We will listen to your opinions through any alternatives to speech you may choose to use. Your carer/loved one is also very welcome to accompany you and be your voice. We have included a sheet with a schedule of the meeting and an outline of what we are going to discuss together. You can prepare some responses in advance if that is helpful.

The meeting will be a small, friendly gathering of up to 20 people, all from the MND community of patients, relatives, carers and health-care professionals, hosted by the MND service at Prospect and supported by the MND Association. It will be recorded for research and will not be shared outside of that purpose; data will be treated anonymously according to the General Data Protection Regulation. You will be contacted on the phone by a member of the team to hear if you are able to attend and to check for dietary requirements.

We hope you will join us.

**Sara Mazzucco**	**Dorinda Moffatt**

Consultant Neurologist	MND and Neurorespiratory

Great Western Hospital Foundation Trust	Specialist Practitioner

**Chris Bennett**	**Lucie Spurway**

Head of Regional Care Partnerships	Specialist Speech and Language

Central and West	Therapist.

Motor Neurone Disease Association

MND association

PROSPECT Hospice

The Great Western Hospital NHS Foundation Trust

**‘Treatment of choking in people living with Motor Neurone Disease:**

**what is missing?’**

**February 10th 2024 10.30 am to 12.30 pm**

**Prospect Hospice, Moormead Road, Wroughton, Swindon SN4 9BY**

- *Parking available*
- *Lunch provided*
- *Travel expense reimbursement available*

**MEETING AGENDA**

10.30: Welcome and introductions (*Chris Bennett*).

11.00: Scope of the meeting: ‘Why should we talk about choking in people living with MND?’ (presented by *Dorinda Moffatt*) and Q&A

11.20: Break

11.30: Brainstorming session (chaired by *Sara Mazzucco*) on:

1. What about choking matters most to you?

2. How would you like to see choking episodes treated?

3. What needs to change to improve treatment of choking?

4. What is the best way to measure how well choking is treated?

12.00: Discussion (facilitated by *Lucie Spurway, Dorinda Moffatt and Sara Mazzucco*)

12.30: Buffet lunch

**Patient and Public Involvement meeting on:**

**‘Treatment of choking in people living with Motor Neurone Disease:**

**what is missing?’**

***February 10^th^ 2024 10.30 am to 12.30 pm***

***Prospect Hospice, Moormead Road, Wroughton, Swindon SN4 9BY***

I consent for the meeting to be audio-recorded for research purposes. I understand that the content will be treated anonymously according to the General Data Protection Regulation.

Signed	Date

…………………………………….. …………………………..

**Patient and Public Involvement meeting**

**‘Treatment of choking in people living with Motor Neurone Disease:**

**what is missing?’**

***February 10^th^ 2024 10.30 am to 12.30 pm, Prospect Hospice, Swindon***

**Feedback form**

How relevant do you think the topic of this meeting was?

O Not at all

O Neutral

O Quite relevant

O Very relevant

Comment: ………………………………………………………………………………………………………………………………………………………………………………………………………………………………………………………………………………………………………………………………………………………………………

How did you find the format of the meeting?

O Not appropriate

O Neutral

O Appropriate

O Good

Comment:

………………………………………………………………………………………………………………………………………………………………………………………………………………………………………………………………………………………………………………………………………………………………………

Please feel free to add here any other comment and/or suggestion about the meeting

………………………………………………………………………………………………………………………………………………………………………………………………………………………………………………………………………………………………………………………………………………………………………

**PPI co-design meeting participants**

| **Participant** | **Role** | **Communication requirements** | **Communication aids** | **Sex assigned at birth** | **Additional information** |
| --- | --- | --- | --- | --- | --- |
| Participant A | plwMND | Anarthria | Head-mouse operated speech generating device | Male | King’s Stage 4 |
| Participant B | plwMND | Disarthria | no | Male | King’s Stage 2 |
| Participant C | Healthcare professional |  | | Female | Neurorespiratory Specialist Practitioner |
| Participant D | Healthcare professional |  | | Female | Healthcare assistant |
| Participant E | Healthcare professional |  | | Female | Specialist Speech and Language Therapist |
| Participant F | Carer |  | | Female | Participant A’s wife |
| Participant G | Carer |  | | Female | Participant B’s wife |
| Participant H | Carer |  | | Female | Participant B’s daughter |
| Participant I | Carer |  | | Female | Participant B’s daughter |
| Participant L | Family member |  | | Male | Participant B’s family member |
| Participant M | Carer |  | | Male | Husband of a deceased patient |
| Participant N | Carer |  | | Male | Son of a deceased patient |
| Participant O | Carer |  | | Female | Partner of a deceased patient |
| Participant P | Funder |  | | Male | Representative of the MND Association |
| Participant Q | Chair |  | | Female | Neurology consultant |

**Patient and Public Involvement meeting report:**

**‘Treatment of choking in people living with Motor Neurone Disease:**

**what is missing?’**

February 10^th^ 2024 10.30 am to 12.30 pm

Prospect Hospice, Moormead Road, Wroughton, Swindon SN4 9BY

This event, funded by the Motor Neurone Disease Association (MNDA) and hosted by PROSPECT Hospice, was born from the partnership between The Great Western Hospital NHS Foundation Trust, the MNDA and Prospect Hospice, through a group of clinicians leading the clinical service for people living with MND in the Swindon area.

The occasion was offered by the upcoming opportunity to apply for funding within the ‘Healthcare’ research stream of the MNDA. The group had clinically identified a knowledge gap and an unmet need around management of choking in people living with MND. The purpose of the Patient and Public involvement meeting was to explore views, values and priorities of people with lived experience of MND, to inform a Healthcare project to submit to the MNDA bid. For the purpose of this report, this project will be called ‘Improving choking in MND’ project.

The meeting was designed and organised by Sara Mazzucco, consultant neurologist and MND lead for the Great Western Hospital, Dorinda Moffatt, MND and Neurorespiratory Specialist Practitioner and MND coordinator for Swindon, Chris Bennett, Head of Regional Care, Central and West Partnerships, Motor Neurone Disease Association, and Lucie Spurway, Specialist Speech and Language Therapist at the Great Western Hospital, with the aid of Kerry Parkes, MND healthcare assistant and Samantha Bennetta, administrative team leader, and with the support of Carolyn Bell, director of Patients services at Prospect Hospice.

The venue was kindly provided free of charge by the Hospice in a large and airy room with all the necessary facilities, and refreshments and travel expenses for invited participants were funded by the MNDA.

A sample of people with lived experience of MND, both patients, carers and relatives known to the clinical service was invited, aiming to be representative of the population at high risk of choking and their carers. A written invitation with a schedule for the morning was sent out, to allow patients with communication difficulties to get prepared in advance if they wanted, or to bring with them their communication aids. This was followed-up with a phone call by a member of the team to answer any question and offer clarification when needed.

Two patients with MND accepted the invitation. One is a young man of forty-seven year with advanced disease (Patient A), quadriplegia and anarthria, who used his own gaze-operated communication aid during the meeting; he came on his wheel chair with a headrest accompanied by his wife. Lucy Spurway helped with the set-up of his communication aid and supported him throughout the meeting to allow him full participation. The other patient (Patient B) is an eighty-year old gentlemen with a bulbar form of MND and moderate to severe dysarthria, who just underwent his voice-banking process; he came wearing his head-up collar accompanied by his wife, two daughters and one son in law. The remaining participants were the spouses and one son of two patients recently deceased due to the disease. The organisers were all presents.

The invitation letter and the morning schedule are attached.

Informed consent forms to audio-recording were signed by all participants. Refreshments on arrival were provided.

The meeting started with Dorinda Moffatt introducing the plan for the morning and the scope of the meeting, in particular defining the concept of ‘choking’ in MND and its differences to the general population, and the gap in existing knowledge and available training on how to treat choking in people living with MND. Chris Bennet welcomed everyone on behalf of the MNDA and a round of introductions followed. Participants were advised that, to allow participation of the two patients, both with communication problems, each patient would be given time to formulate their contribution at the time they wished to offer it, and that the discussion would pause until the contribution was completed.

After a short break and the opportunity for more refreshments, Sara Mazzucco introduced the brainstorming session, based on the model of the ‘theory of change’ (TOC). Based on the assumptions illustrated by Dorinda Moffatt on the gap of existing knowledge, the group agreed that there is substantial scope for improvement of the management of choking. This was chosen as long-term outcome of the ‘Improving choking in MND’ project.

According to the TOC, this broad outcome was broken into overarching sets of more specific outcomes (Figure 1, Outcomes 1,2,3,4); for each, a number of ‘Prerequisites’ were defined; these were intended as the ‘ingredients’ that the group thought would be necessary to achieve the long-term outcome. For each of them, a number of ‘Interventions’ were identified, each in turn with a suggested ‘Indicator’ as a measure of the achieved change (Figure 1).

The first set of outcomes identified was an overwhelming, unmet need for more information on choking, early after the diagnosis of MND; patients expressed the need to be educated on the causes of choking and on how to manage it. They unanimously asked for evidence-based, clear and accessible guidance through episodes of choking. In their words ‘*guidance needs to be laminated and hung on the fridge*.’ However there was agreement that patients should be respected in their wishes on when to be informed about what to expect from the disease, and communication should be delivered in a tailored way depending on patients wishes and circumstances. A relative highlighted how there is a need for dedicated staff to allow for these conversations to happen timely. Patient B voiced his frustration for not enough research being devolved to health care delivery. Patient A highlighted the importance of having access to functioning equipment and medication in a timely way. Interventions and indicators are summarised in Figure 1.

The second set of outcomes was access to and guidance for medications used for management of choking. The medication of choice, the dose, formulation and route of administration need to be specified in detail in a flow chart easy to understand on which patients and carers need to be trained. There was consensus on the need of such guidance and on the need to have it early on in the course of the disease. Interventions and indicators are summarised in Figure 1.

The third set of outcomes converged on equipment used both as a means of treatment and prevention of choking and on correct positioning of patients as suggested by Physiotherapy and Occupational Therapy. There is concern around long waiting times to access life-changing aids such as cough-assist machines that help with airways clearing. Patient A shared his experience of having to wait nearly one year to have a cough-assist machine. There is sometime confusion on which should be the provider of some pieces of equipment such as neck collars (whether the Community or the Trust and what department in the Trust, whether Neurology or Physiotherapy). Accountability and agreed pathways, together with adequate funding, can expedite access to much needed equipment. As every palliative measure aiming to improve quality of life, timing is critical and delays should be avoided (see Interventions and indicators are summarised in Figure 1).

The fourth and last outcome was simply the unanimous agreement and request from patients and carers to be in a position to treat choking at home, without anxiety-generating calls to 111, the GP, the MND coordinator or the Neurology consultant. Patients want to be kept out of hospital and to be empowered to treat choking episodes at home on their own. This would also benefit the NHS, sparing emergency consultations, ambulances attendance, and hospital admissions.

Lastly, when asked how patients should be approached to be enrolled in the Improving choking in MND project, they asked the approach to be individualised; patient A preferred at his current stage to have email interviews with a list of questions and space for comments, while Patient B preferred a face-to-face individual interview. Carers and relatives agreed that they would be happy with a group discussion such as a focus group. There was an agreement that patients and carers should be interviewed separately to allow to freely express their emotions.

Sara Mazzucco, Dorinda Moffatt, Chris Bennet and Lucie Spurway facilitated the discussion and helped to crystallise the preconditions, interventions and indicators, on which full agreement was sought. The brainstorming overran by 10 minutes due to the large contribution of participants, including the two patients. Anonymous feedback forms were given out and completed by all participants.

To the feedback question: ‘How relevant do you think the topic of this meeting was?’ 100% of participants responded ‘Very relevant’. To the question: ‘How did you find the meeting format?’ 25% responded ‘Appropriate’, 75% responded ‘Good’. Comments were left on how the meeting was ‘Open, friendly and informative’ ‘interesting and helpful’ ‘with interesting discussion -lots of audience participation’, and that it ‘Raised very important issues’ and ‘everyone had opportunity to speak’. Two comments were made on the importance for patients to have appropriate information either in written or in person early on in the course of the disease, and also on the importance of educating GP who often ‘have no experience of the disease’.

All participants gathered for lunch.

This meeting was important and, in some respects, unique because:

- It was born out of a collaboration between an NHS Trust, the Community and a Patients’ Association
- It was highly efficient, collecting a wealth of information from patients and carers in a very short time (just over an hour of brainstorming and discussion)
- It involved patients with lived experience of the disease with severe communication impairment, facilitating their participation in full and proving that involvement of such patients is not only possible, but highly beneficial for research given the amount of information they are willing to share
- It will inform a research project on healthcare delivery for people living with MND, and it has the potential to change clinical practice on the basis of the feedback received
- While highlighting unmet needs for people living with MND and their carers, it also outlines interventions with the potential to save money to the NHS.
